# Supplementary material for: Rapid spread of a densovirus in a major crop pest following wide-scale adoption of Bt-cotton in China
Source: eLife. 2021 Jul 15;10:e66913. doi: 10.7554/eLife.66913 (PMC8324301; doi:10.7554/eLife.66913)
Supplement: Figure 1—source data 1. [file elife-66913-fig1-data1.docx]

Source data for figure 1

| Strain | LC_50_^b^ (HaDV2^_^)^c^ | LC_50_^b^ (HaDV2+)^c^ |
| --- | --- | --- |
| 96S | 0.04 | 0.06 |
| LF | 0.04 | 0.06 |
| LF5 | 3.4 | 4.72 |
| LF60 | 16.1 | 26.3 |
| F120 | 26.6 | 34.51 |
| LF240 | 47.5 | 61.82 |
| LFC2 | 10.33 | 16.4 |
| 96CAD | 7.12 | 12.1 |
| BtR | 11.91 | 27.3 |
