# Supplementary material for: Rapid spread of a densovirus in a major crop pest following wide-scale adoption of Bt-cotton in China
Source: eLife. 2021 Jul 15;10:e66913. doi: 10.7554/eLife.66913 (PMC8324301; doi:10.7554/eLife.66913)
Supplement: Figure 1—figure supplement 1—source data 1. [file elife-66913-fig1-figsupp1-data1.docx]

Source data for figure1- figure supplement 1

**Quantification of HaDV2 in individuals feeding on diet with and without Bt toxin.**

| Diet | Body weight (g) | Volume of DNA samples (µL) | Ct Value | Copies/individual |
| --- | --- | --- | --- | --- |
| Non Bt | 0.0201 | 500 | 34.239 | 39603.93838 |
| Non Bt | 0.0239 | 333.3333333 | 37.495 | 2457.680709 |
| Non Bt | 0.0085 | 200 | 35.67 | 5579.871526 |
| Non Bt | 0.0253 | 500 | 37.575 | 3477.619282 |
| Non Bt | 0.0116 | 100 | 35.5 | 3158.132947 |
| Non Bt | 0.0216 | 200 | 36.509 | 3026.402567 |
| Non Bt | 0.0107 | 200 | 36.175 | 3860.99291 |
| Non Bt | 0.0156 | 333.3333333 | 35.835 | 8245.560121 |
| Non Bt | 0.0269 | 500 | 36.353 | 8477.53009 |
| Non Bt | 0.0179 | 200 | 34.706 | 11269.56485 |
| Non Bt | 0.0033 | 100 | 33.119 | 17924.78495 |
| Non Bt | 0.0124 | 200 | 34.8 | 10522.98358 |
| Non Bt | 0.0224 | 100 | 34.265 | 7772.032345 |
| Non Bt | 0.0147 | 100 | 33.351 | 15135.03289 |
| Non Bt | 0.017 | 100 | 33.943 | 9828.940459 |
| Non Bt | 0.0155 | 100 | 34.231 | 7967.128837 |
| Non Bt | 0.0191 | 100 | 33.192 | 16995.58251 |
| Bt | 0.0012 | 100 | 34.251 | 7851.780842 |
| Bt | 0.0013 | 100 | 34.007 | 9380.780124 |
| Bt | 0.0017 | 100 | 37.648 | 659.4686144 |
| Bt | 0.0016 | 100 | 35.481 | 3202.192205 |
| Bt | 0.0013 | 100 | 37.087 | 992.7813405 |
| Bt | 0.0023 | 100 | 37.611 | 677.5033124 |
| Bt | 0.001 | 100 | 36.934 | 1109.957091 |
| Bt | 0.0009 | 100 | 35.162 | 4040.819489 |
| Bt | 0.001 | 100 | 37.095 | 987.0067835 |
| Bt | 0.0011 | 100 | 35.817 | 2506.350019 |
| Bt | 0.0012 | 100 | 35.515 | 3123.77795 |
| Bt | 0.0012 | 100 | 34.849 | 5076.815761 |
| Bt | 0.0016 | 100 | 35.951 | 2273.03501 |
| Bt | 0.0004 | 100 | 36.153 | 1961.715605 |
| Bt | 0.0016 | 100 | 35.664 | 2802.168878 |
| Bt | 0.0008 | 100 | 37.567 | 699.5930708 |
| Bt | 0.0009 | 100 | 34.722 | 5569.423135 |
