# Supplementary material for: Rapid spread of a densovirus in a major crop pest following wide-scale adoption of Bt-cotton in China
Source: eLife. 2021 Jul 15;10:e66913. doi: 10.7554/eLife.66913 (PMC8324301; doi:10.7554/eLife.66913)
Supplement: Figure 2—source data 1. [file elife-66913-fig2-data1.docx]

|  | Larval period (days) | Stand Error | Significance level | Female pupa period (days) | Stand Error | Significance level | Male pupa period (days) | Stand Error | Significance level |
| --- | --- | --- | --- | --- | --- | --- | --- | --- | --- |
| LF D+ | 16.76 | 0.2400 | f | 11.23 | 0.1747 | e | 13.03 | 0.3645 | e |
| LF D- | 17.43 | 0.1871 | e | 11.94 | 0.2206 | cd | 13.78 | 0.3109 | c |
| LF60 D+ | 17.74 | 0.1404 | de | 11.73 | 0.2145 | de | 13.60 | 0.3278 | cd |
| LF60 D- | 18.20 | 0.1051 | c | 12.41 | 0.1955 | bc | 14.28 | 0.2883 | ab |
| LF5 D+ | 17.38 | 0.1362 | e | 11.58 | 0.2404 | de | 13.29 | 0.2365 | de |
| LF5 D- | 17.92 | 0.1065 | cd | 12.08 | 0.2205 | cd | 13.63 | 0.2535 | cd |
| LF240 D+ | 19.03 | 0.1454 | b | 12.93 | 0.1498 | ab | 14.23 | 0.2665 | b |
| LF240 D- | 19.61 | 0.1705 | a | 13.31 | 0.1430 | a | 14.68 | 0.2478 | a |
