# Supplementary material for: Rapid spread of a densovirus in a major crop pest following wide-scale adoption of Bt-cotton in China
Source: eLife. 2021 Jul 15;10:e66913. doi: 10.7554/eLife.66913 (PMC8324301; doi:10.7554/eLife.66913)
Supplement: Figure 5—source data 1. [file elife-66913-fig5-data1.docx]

|  | XJ_Infection_rate | AC_Infection_rate | XJ_RADR | Standard error | AC_RADR | Standard error |
| --- | --- | --- | --- | --- | --- | --- |
| 2007 | 0.43333333 | 0.36666667 | 0.51583016 | 0.00228193 | 0.50184658 | 0.00344385 |
| 2008 | 0.51190476 | 0.59302326 | 0.53112553 | 0.00279861 | 0.50380542 | 0.00221757 |
| 2009 | 0.83838384 | 0.71929825 | 0.62165876 | 0.00348581 | 0.54466629 | 0.00219933 |
| 2010 | 0.95145631 | 0.63636364 | 0.59262144 | 0.00185859 | 0.58178999 | 0.00246084 |
| 2011 | 0.82989691 | 0.96938776 | 0.58994213 | 0.00431552 | 0.59676126 | 0.00567597 |
| 2012 | 0.85502959 | 0.86394558 | 0.58109063 | 0.00331075 | 0.52557795 | 0.00370361 |
| 2013 | 1 | 1 | 0.6209697 | 0.00211283 | 0.61752387 | 0.00421606 |
| 2014 | 1 | 0.93055556 | 0.66278689 | 0.00665795 | 0.61731615 | 0.01657937 |
| 2015 | 0.97701149 | 0.97641509 | 0.58722949 | 0.00318613 | 0.6707153 | 0.00437566 |
| 2016 | 0.96715328 | 0.96470588 | 0.60662197 | 0.00899517 | 0.61649216 | 0.00715627 |
