# Supplementary material for: Rapid spread of a densovirus in a major crop pest following wide-scale adoption of Bt-cotton in China
Source: eLife. 2021 Jul 15;10:e66913. doi: 10.7554/eLife.66913 (PMC8324301; doi:10.7554/eLife.66913)
Supplement: Supplementary file 1. — (a) Bt toxin sensitivity test of different Helicoverpa armigera strains with or without HaDV2 infection. (b) Mortality changes with Bt toxin concentration test of different H. armigera strains with or without HaDV2 infection. (c) Comparing the effects of HaDV2 on fitness components of LF, LF5, LF60, and LF240. LF is susceptible strain; LF5, LF60, and LF240 are Bt resistant strains selected with LF strain. Significant differences (ANOVA followed by Tukey’s HSD test) between each strain with or without HaDV2 infestation are indicated by different letters (p<0.05). Insects were reared on artificial non-Bt-diet. D+ stand for infected by HaDV2, D− stand for non-infected by HaDV2. (1–5) means the survival rate from the first star to the 5th star; 5 p: from the 5th star to pupa; Proportion FA: the rate of female divided male. (d) Analysis of variance for fitness parameters of 4 cotton bollworm strains (LF, LF5, LF60, and LF240). (e) Fitness of the susceptible and resistant strains on Bt- and non-Bt-cotton infected with or without HaDV2. (f) Analysis of variance for weight of cotton bollworm larvae. (g) Sample information and infection rates of HaDV2 in the field populations of H. armigera. See Figure 6 for a map of locations. X: east longitude, Y: northern latitude. (h) Host information and accessions for samples. [file elife-66913-supp1.docx]

**Supplementary File 1**

**Rapid spread of a symbiotic virus in a major crop pest following wide-scale adoption of Bt-cotton in China**

Yutao Xiao^1,2^^†^, Wenjing Li^1,3†^, Xianming Yang^1†^, Pengjun Xu^4,5†^, Minghui Jin^2^, He Yuan^2^, Weigang Zheng^2^, Mario Soberón^6^, Alejandra Bravo^6^, Kenneth Wilson^5,2^, Kongming Wu^1,*^

**Supplementary file 1a.** **Bt toxin sensitivity test of different *H. armigera* strains with or without HaDV2 infection**

| Strain^a^ | LC_50_^b^ (HaDV2^_^)^c^  (95% fiducial limits)  (µg Cry1Ac per gr diet) | Resistant times | LC_50_ (HaDV2+)  (95% fiducial limits)  (µg Cry1Ac per gr diet) | Resistant times | Ratio of resistant times of the same strain when infected with or without HaDV2 |
| --- | --- | --- | --- | --- | --- |
| 96S | 0.04(0.01-0.09) | 1 | 0.06(0.003-0.22) | 1.5 | 1.5 |
| LF | 0.04(0.02-0.06) | 0.98 | 0.06(0.04-0.09) | 1.5 | 1.5 |
| LF5 | 3.40(2.0-5.6) | 85 | 4.72(2.9-8.5) | 118 | 1.4 |
| LF60 | 16.1(3.3-56) | 403 | 26.3(17-41) | 658 | 1.6 |
| F120 | 26.6(0.01-167) | 665 | 34.51(0.53-118) | 863 | 1.3 |
| LF240 | 47.5(5.8-667) | 1188 | 61.82(11-694) | 1546 | 1.3 |
| LFC2 | 10.33(1.7-32) | 258 | 16.4(10-26) | 410 | 1.6 |
| 96CAD | 7.12 (0.44-25) | 178 | 12.1(7.0-19) | 303 | 1.7 |
| BtR | 11.91(0.30-63) | 298 | 27.3 (18-41) | 683 | 2.3 |

^a^: 96S and LF are two Bt-susceptible strains; BtR and 96CAD are two Bt-resistant strains selected with 96S; LF5, LF60, LF120, LF240 and LFC2 are Bt-resistant strains selected with the LF strain. The detail of these strains is described in the Methods section. ^b^: LC_50_ of all the strains are divided by LC_50_ of 96S. ^c^: Infected with HaDV2 (HaDV2+) and not infected with HaDV2 (HaDV2-).

**Supplementary file 1b. Mortality changes with Bt toxin concentration test of different *H. armigera* strains with or without HaDV2 infection.**

| Strain | concentration | HaDV2+ | | HaDV2- | |
| --- | --- | --- | --- | --- | --- |
|  | Cry1Ac(μg/ml) | total pest | mortality | total pest | mortality |
| 96s | 1.000 | 72 | 56 | 72 | 69 |
|  | 0.500 | 72 | 50 | 72 | 59 |
|  | 0.125 | 72 | 45 | 72 | 50 |
|  | 0.031 | 72 | 38 | 72 | 39 |
|  | 0.016 | 72 | 18 | 72 | 20 |
|  | 0.000 | 72 | 1 | 72 | 5 |
| LF | 1.000 | 46 | 44 | 47 | 46 |
|  | 0.500 | 48 | 36 | 47 | 40 |
|  | 0.125 | 48 | 31 | 47 | 34 |
|  | 0.031 | 45 | 18 | 46 | 23 |
|  | 0.016 | 47 | 12 | 48 | 14 |
|  | 0.000 | 48 | 6 | 48 | 6 |
| LF5 | 16.000 | 70 | 66 | 72 | 72 |
|  | 8.000 | 72 | 41 | 69 | 54 |
|  | 4.000 | 72 | 30 | 72 | 33 |
|  | 2.000 | 72 | 18 | 70 | 21 |
|  | 1.000 | 72 | 7 | 72 | 11 |
|  | 0.000 | 72 | 0 | 72 | 4 |
| LF60 | 128.000 | 72 | 54 | 72 | 65 |
|  | 64.000 | 72 | 47 | 72 | 51 |
|  | 32.000 | 72 | 32 | 72 | 37 |
|  | 8.000 | 72 | 21 | 72 | 23 |
|  | 1.000 | 72 | 13 | 72 | 13 |
|  | 0.000 | 72 | 0 | 72 | 5 |
| LF120 | 256.000 | 72 | 69 | 72 | 71 |
|  | 128.000 | 72 | 60 | 72 | 59 |
|  | 64.000 | 72 | 38 | 72 | 39 |
|  | 32.000 | 72 | 23 | 72 | 29 |
|  | 1.000 | 72 | 6 | 72 | 8 |
|  | 0.000 | 72 | 3 | 72 | 4 |
| LF240 | 256.000 | 48 | 45 | 48 | 46 |
|  | 128.000 | 72 | 47 | 72 | 51 |
|  | 64.000 | 72 | 24 | 72 | 27 |
|  | 8.000 | 72 | 12 | 72 | 14 |
|  | 1.000 | 72 | 2 | 72 | 4 |
|  | 0.000 | 72 | 3 | 72 | 5 |
| LFC2 | 128.000 | 47 | 40 | 46 | 42 |
|  | 64.000 | 48 | 33 | 48 | 36 |
|  | 32.000 | 48 | 26 | 46 | 27 |
|  | 8.000 | 48 | 15 | 47 | 18 |
|  | 1.000 | 48 | 10 | 48 | 12 |
|  | 0.000 | 48 | 2 | 47 | 5 |
| 96CAD | 128.000 | 48 | 41 | 46 | 44 |
|  | 64.000 | 48 | 35 | 46 | 38 |
|  | 32.000 | 48 | 30 | 46 | 30 |
|  | 8.000 | 46 | 16 | 47 | 19 |
|  | 1.000 | 48 | 11 | 48 | 14 |
|  | 0.000 | 48 | 4 | 48 | 5 |
| BtR | 256.000 | 48 | 44 | 48 | 45 |
|  | 128.000 | 48 | 33 | 48 | 34 |
|  | 64.000 | 48 | 31 | 48 | 32 |
|  | 8.000 | 47 | 11 | 48 | 17 |
|  | 1.000 | 47 | 5 | 48 | 14 |
|  | 0.000 | 47 | 1 | 47 | 8 |

**Supplementary file 1c. Comparing of the effects of HaDV2 on fitness components of LF, LF5, LF60 and LF240.** LF is susceptible strain; LF5, LF60 and LF240 are Bt resistant strains selected with LF strain. Significant differences (ANOVA followed by Tukey’s HSD test) between each strain with or without HaDV2 infestation are indicated by different letters (p < 0.05). Insects were reared on artificial non Bt-diet. D+ stand for infected by HaDV2, D- stand for non-infected by HaDV2. (1-5) means the survival rate from the first star to the 5th star; 5-p: from the 5th star to pupa; Proportion FA: the rate of female divided male.

| *H. armigera* strains | Survival rate (1-5) | Survival rate (5-p) | Larval period (days) | Pupal weight  (mg) | Female pupa period (days) | Male pupa period (days) | Emergence rate | Proportion FA | Longevity of female adult | Longevity of male adult | Number of eggs produced per female | Hatching rate |
| --- | --- | --- | --- | --- | --- | --- | --- | --- | --- | --- | --- | --- |
| LF D+ | 0.92±0.02 a | 0.94±0.03 a | 16.76±0.24 f | 236.11±9.55 a | 11.23±0.17 e | 13.03±0.36 e | 0.92±0.032 a | 0.51±0.04 a | 8.07±0.33 a | 9.52±0.27 abc | 477.29±10.46 a | 0.69±0.02 a |
| LF D- | 0.89±0.02 abc | 0.91±0.03 abc | 17.43±0.19 e | 224.68±21.61 ab | 11.94±0.22 cd | 13.78±0.31 c | 0.92±0.024 a | 0.51±0.05 a | 7.61±0.34 a | 9.92±0.40 ab | 402.17±11.51 b | 0.62±0.02 bc |
| LF5 D+- | 0.90±0.02 ab | 0.94±0.03 a | 17.38±0.14 e | 223.27±8.02 ab | 11.58±0.24 de | 13.29±0.24 de | 0.92±0.017 a | 0.49±0.02 a | 8.10±0.25 a | 10.33±0.32 a | 408.61±17.23 b | 0.64±0.02 ab |
| LF5 D- | 0.87±0.02 abcd | 0.92±0.02 bc | 17.92±0.11 cd | 215.25±5.70 b | 12.08±0.22 cd | 13.63±0.25 cd | 0.91±0.024 a | 0.50±0.02 a | 7.74±0.27 a | 9.45±0.34 abc | 348.77±12.81 c | 0.59±0.02 bc |
| LF60 D+ | 0.85±0.02 abcd | 0.85±0.03 abc | 17.74±0.14 de | 222.45±6.33 ab | 11.73±0.21 de | 13.60±0.33 cd | 0.91±0.031 a | 0.50±0.02 a | 7.79±0.32 a | 9.14±0.26 bc | 408.32±18.51 b | 0.63±0.02 bc |
|  |  |  |  |  |  |  |  |  |  |  |  |  |
| LF60 D- | 0.81±0.03 cd | 0.85±0.03 bc | 18.20±0.11 c | 224.34±6.42 ab | 12.41±0.20 bc | 14.28±0.29 b | 0.91±0.023 a | 0.49±0.03 a | 7.63±0.33 a | 8.95±0.30 c | 333.45±11.98 c | 0.58±0.02 c |
| LF240 D+ | 0.83±0.03 bcd | 0.83±0.04 c | 19.03±0.15 b | 151.13±4.15 bc | 12.93±0.15 ab | 14.23±0.27 b | 0.85±0.029 b | 0.49±0.02 a | 7.83±0.25 a | 8.76±0.31 c | 340.12±12.30 c | 0.50±0.02 d |
| LF240 D- | 0.80±0.05 d | 0.83±0.04 c | 19.61±0.17 a | 126.25±4.38 c | 13.31±0.14 a | 14.68±0.25 a | 0.80±0.045 b | 0.51±0.03 a | 7.43±0.21 a | 8.57±0.31 c | 292.30±9.52 c | 0.42±0.02 e |

**Supplementary file 1d. Analysis of variance for fitness parameters of 4 cotton bollworm strains (LF, LF5, LF60, and LF240)**

| Source | Dependent variable | Type III sum of squares | df | Mean square | F | P |
| --- | --- | --- | --- | --- | --- | --- |
| Model | Survival rate (1-5) | 0.118 | 12 | 0.010 | 2.336 | 0.025 |
|  | Survival rate (5-p) | 0.157 | 12 | 0.013 | 2.977 | 0.006 |
|  | Larval period (days) | 7853.346 | 25 | 314.134 | 1.233 | 0.205 |
|  | Pupal weight | 28274.921 | 273 | 103.571 | 0.999 | 0.607 |
|  | Female pupa period (days) | 2235.348 | 24 | 93.139 | 0.714 | 0.836 |
|  | Male pupa period (days) | 2241.381 | 21 | 106.732 | 0.868 | 0.633 |
|  | Emergence rate | 0.203 | 12 | 0.017 | 6.835 | 0.000 |
|  | Proportion FA | 0.019 | 12 | 0.002 | 0.285 | 0.988 |
|  | Longevity of female adult | 187.565 | 48 | 3.908 | 1.218 | 0.170 |
|  | Longevity of male adult | 307.072 | 50 | 6.141 | 1.615 | 0.009 |
|  | Number of eggs produced per female | 1148918.561 | 44 | 26111.785 | 4.302 | 0.000 |
|  | Hatching rate | 1.889 | 39 | 0.048 | 4.840 | 0.000 |
| Intercept | Survival rate (1-5) | 35.477 | 1 | 35.477 | 8455.099 | 0.000 |
|  | Survival rate (5-p) | 37.375 | 1 | 37.375 | 8519.698 | 0.000 |
|  | Larval period (days) | 55338.987 | 1 | 55338.987 | 217.264 | 0.000 |
|  | Pupal weight | 86529.972 | 1 | 86529.972 | 834.694 | 0.000 |
|  | Female pupa period (days) | 28229.588 | 1 | 28229.588 | 216.492 | 0.000 |
|  | Male pupa period (days) | 22789.725 | 1 | 22789.725 | 185.415 | 0.000 |
|  | Emergence rate | 38.149 | 1 | 38.149 | 15381.938 | 0.000 |
|  | Proportion FA | 12.070 | 1 | 12.070 | 2145.183 | 0.000 |
|  | Longevity of female adult | 15159.147 | 1 | 15159.147 | 4724.650 | 0.000 |
|  | Longevity of male adult | 23454.485 | 1 | 23454.485 | 6169.256 | 0.000 |
|  | Number of eggs produced per female | 33712416.161 | 1 | 33712416.161 | 5554.433 | 0.000 |
|  | Hatching rate | 74.448 | 1 | 74.448 | 7440.300 | 0.000 |
| RL | Survival rate (1-5) | 0.074 | 3 | 0.025 | 5.850 | 0.002 |
|  | Survival rate (5-p) | 0.091 | 3 | 0.030 | 6.913 | 0.001 |
|  | Larval period (days) | 2216.175 | 9 | 246.242 | 0.967 | 0.467 |
|  | Pupal weight | 27873.003 | 269 | 103.617 | 1.000 | 0.607 |
|  | Female pupa period (days) | 870.277 | 6 | 145.046 | 1.112 | 0.355 |
|  | Male pupa period (days) | 484.192 | 6 | 80.699 | 0.657 | 0.685 |
|  | Emergence rate | 0.079 | 3 | 0.026 | 10.597 | 0.000 |
|  | Proportion FA | 0.002 | 3 | 0.001 | 0.105 | 0.957 |
|  | Longevity of female adult | 2.135 | 3 | 0.712 | 0.222 | 0.881 |
|  | Longevity of male adult | 76.201 | 3 | 25.400 | 6.681 | 0.000 |
|  | Number of eggs produced per female | 548157.882 | 3 | 182719.294 | 30.105 | 0.000 |
|  | Hatching rate | 1.159 | 3 | 0.386 | 38.595 | 0.000 |
| HaDV2 | Survival rate (1-5) | 0.011 | 1 | 0.011 | 2.729 | 0.108 |
|  | Survival rate (5-p) | 0.002 | 1 | 0.002 | 0.473 | 0.496 |
|  | Larval period (days) | 1569.766 | 3 | 523.255 | 2.054 | 0.106 |
|  | Pupal weight | 0.000 | 0 | 0 | 0 | 0 |
|  | Female pupa period (days) | 175.156 | 3 | 58.385 | 0.448 | 0.719 |
|  | Male pupa period (days) | 552.641 | 3 | 184.214 | 1.499 | 0.215 |
|  | Emergence rate | 0.003 | 1 | 0.003 | 1.016 | 0.320 |
|  | Proportion FA | 2.083E-6 | 1 | 2.083E-6 | 0.000 | 0.985 |
|  | Longevity of female adult | 3.424 | 1 | 3.424 | 1.067 | 0.303 |
|  | Longevity of male adult | 3.105 | 1 | 3.105 | 0.817 | 0.367 |
|  | Number of eggs produced per female | 291274.783 | 1 | 291274.783 | 47.990 | 0.000 |
|  | Hatching rate | 0.243 | 1 | 0.243 | 24.301 | 0.000 |
| RL*HaDV2 | Survival rate (1-5) | 0.000 | 3 | 5.092E-5 | 0.012 | 0.998 |
|  | Survival rate (5-p) | 0.001 | 3 | 0.000 | 0.072 | 0.975 |
|  | Larval period (days) | 2494.480 | 12 | 207.873 | 0.816 | 0.634 |
|  | Pupal weight | 0.000 | 0 | 0 | 0 | 0 |
|  | Female pupa period (days) | 1189.612 | 14 | 84.972 | 0.652 | 0.820 |
|  | Male pupa period (days) | 1336.922 | 11 | 121.538 | 0.989 | 0.457 |
|  | Emergence rate | 0.004 | 3 | 0.001 | 0.584 | 0.630 |
|  | Proportion FA | 0.002 | 3 | 0.001 | 0.095 | 0.962 |
|  | Longevity of female adult | 1.287 | 3 | 0.429 | 0.134 | 0.940 |
|  | Longevity of male adult | 19.051 | 3 | 6.350 | 1.670 | 0.174 |
|  | Number of eggs produced per female | 17392.772 | 3 | 5797.591 | 0.955 | 0.415 |
|  | Hatching rate | 0.008 | 3 | 0.003 | 0.282 | 0.839 |
| Error | Survival rate (1-5) | 0.147 | 35 | 0.004 |  |  |
|  | Survival rate (5-p) | 0.154 | 35 | 0.004 |  |  |
|  | Larval period (days) | 101628.692 | 399 | 254.709 |  |  |
|  | Pupal weight | 311.000 | 3 | 103.667 |  |  |
|  | Female pupa period (days) | 36901.857 | 283 | 130.395 |  |  |
|  | Male pupa period (days) | 34415.271 | 280 | 122.912 |  |  |
|  | Emergence rate | 0.087 | 35 | 0.002 |  |  |
|  | Proportion FA | 0.197 | 35 | 0.006 |  |  |
|  | Longevity of female adult | 808.548 | 252 | 3.209 |  |  |
|  | Longevity of male adult | 1037.901 | 273 | 3.802 |  |  |
|  | Number of eggs produced per female | 1414184.518 | 233 | 6069.461 |  |  |
|  | Hatching rate | 2.041 | 204 | 0.010 |  |  |
| Total | Survival rate (1-5) | 35.741 | 48 |  |  |  |
|  | Survival rate (5-p) | 37.685 | 48 |  |  |  |
|  | Larval period (days) | 430366.000 | 425 |  |  |  |
|  | Pupal weight | 117472.000 | 277 |  |  |  |
|  | Female pupa period (days) | 160385.000 | 308 |  |  |  |
|  | Male pupa period (days) | 150869.000 | 302 |  |  |  |
|  | Emergence rate | 38.439 | 48 |  |  |  |
|  | Proportion FA | 12.286 | 48 |  |  |  |
|  | Longevity of female adult | 18955.000 | 301 |  |  |  |
|  | Longevity of male adult | 29513.000 | 324 |  |  |  |
|  | Number of eggs produced per female | 42272760.000 | 278 |  |  |  |
|  | Hatching rate | 88.383 | 244 |  |  |  |
| Corrected total | Survival rate (1-5) | 0.264 | 47 |  |  |  |
|  | Survival rate (5-p) | 0.310 | 47 |  |  |  |
|  | Larval period (days) | 109482.038 | 424 |  |  |  |
|  | Pupal weight | 28585.921 | 276 |  |  |  |
|  | Female pupa period (days) | 39137.205 | 307 |  |  |  |
|  | Male pupa period (days) | 36656.652 | 301 |  |  |  |
|  | Emergence rate | 0.290 | 47 |  |  |  |
|  | Proportion FA | 0.216 | 47 |  |  |  |
|  | Longevity of female adult | 996.113 | 300 |  |  |  |
|  | Longevity of male adult | 1344.972 | 323 |  |  |  |
|  | Number of eggs produced per female | 2563103.079 | 277 |  |  |  |
|  | Hatching rate | 3.930 | 243 |  |  |  |

The categorical variables were RL (resistant levels of different susceptible or resistant strains) and HaDV2 (infected with HaDV2 or not). Interactions were not significant.

**Supplementary file 1e. Fitness of the susceptible and resistant strains on Bt- and non-Bt-cotton infected with or without HaDV2**

| Cotton types | With or without HaDV2 | LF | LF5 | LF 60 | LF120 | LF240 |
| --- | --- | --- | --- | --- | --- | --- |
| Non Bt-cotton | HaDV2+ | 30.95±0.84 ^A a^ | 30.20±0.90 ^A a^ | 25.41±0.97 ^B a^ | 25.00±0.70 ^B a^ | 22.57±0.54 ^C a^ |
|  | HaDV2- | 26.39±0.68 ^A b^ | 24.62±0.66 ^B b^ | 19.79±0.66 ^C b^ | 19.96±0.54 ^C b^ | 17.46±0.53 ^D b^ |
| Bt-cotton | HaDV2+ |  | 28.06±0.72 ^A a^ | 23.71±0.74 ^BC a^ | 24.88±0.66 ^B a^ | 22.33±0.57 ^C a^ |
|  | HaDV2- |  | 22.11±0.73 ^A c^ | 18.81±0.58 ^B b^ | 18.67±0.69 ^B b^ | 16.79±0.49 ^C b^ |

All numbers (mg) are weight after 9 days feeding on Bt-cotton or non Bt-cotton. All strains are described as in Supplementary Table 4. HaDV2+ = infected by HaDV2, HaDV2- = non-infected by HaDV2. Values are means ± SE. Different upper case letters indicate significant differences between *H. armigera* strains for a given treatment. Different lower case letters indicate significant differences between treatments (HaDV2-infected or not) within each strain.

| Source | Type III sum of squares | df | Mean square | F | P |
| --- | --- | --- | --- | --- | --- |
| Model | 18650.995 | 77 | 242.221 | 10.762 | 0.000 |
| Intercept | 335764.047 | 1 | 335764.047 | 14918.250 | 0.000 |
| Bt | 415.442 | 1 | 415.442 | 18.458 | 0.000 |
| RL | 7536.653 | 6 | 1256.109 | 55.810 | 0.000 |
| DNV | 7299.219 | 1 | 7299.219 | 324.310 | 0.000 |
| Bt * RL | 120.643 | 4 | 30.161 | 1.340 | 0.253 |
| Bt * HaDV2 | 0.846 | 1 | 0.846 | .038 | 0.846 |
| RL * HaDV2 | 22.977 | 4 | 5.744 | 0.255 | 0.907 |
| Bt * RL * HaDV2 | 28.605 | 4 | 7.151 | .318 | 0.866 |
| Error | 23519.745 | 1045 | 22.507 |  |  |
| Total | 631863.849 | 1123 | 631863.849 |  |  |
| Corrected total | 42170.740 | 1122 | 42170.740 |  |  |
|  |  |  |  |  |  |

**Supplementary file 1f. Analysis of variance for weight of cotton bollworm larvae**

The categorical variables were Bt (feed on Bt-cotton or non-Bt-cotton), RL (resistant levels of different susceptible or resistant *H. armigera* strains) and HaDV2 (infected with DV2 or not). Red numbers mean significant difference. Interactions were not significant.

**Supplementary file 1g. Sample information and infection rates of HaDV2 in the field populations of *Helicoverpa armigera*.** See Supplementary Fig. 2 for a map of locations. X: [east](file:///E:\program%20files\Youdao\Dict\7.2.0.0703\resultui\dict\?keyword=east) [longitude](file:///E:\program%20files\Youdao\Dict\7.2.0.0703\resultui\dict\?keyword=longitude), Y: northern latitude.

| Population | code | area | Year | n | x | y | HaDV2+ | HaDV2- |
| --- | --- | --- | --- | --- | --- | --- | --- | --- |
| Dezhou, Shandong | 1 | Bt | 2014 | 24 | 115.97 | 36.97 | 24 | 0 |
| Dezhou, Shandong† | 2 | Bt | 2014 | 63 | 115.97 | 36.97 | 63 | 0 |
| Hetian, Xinjiang† | 3 | Bt | 2014 | 22 | 79.91 | 37.23 | 13 | 9 |
| Langfang, Hebei | 4 | Bt | 2014 | 72 | 116.61 | 39.52 | 66 | 6 |
| Luoyang, Henan† | 5 | Bt | 2014 | 24 | 112.68 | 34.66 | 23 | 1 |
| Qianjiang, Hubei† | 6 | Bt | 2014 | 23 | 113.04 | 30.36 | 20 | 3 |
| Shenyang, Liaoning† | 7 | Bt | 2014 | 23 | 123.71 | 41.88 | 19 | 4 |
| Weinan, Shanxi | 8 | Bt | 2014 | 24 | 109.97 | 34.83 | 24 | 0 |
| Xinxiang,Henan | 9 | Bt | 2014 | 21 | 113.81 | 35.16 | 19 | 2 |
| Xinxiang,Henan† | 10 | Bt | 2014 | 24 | 113.94 | 35.12 | 24 | 0 |
| Yantai, Shandong | 11 | Bt | 2014 | 48 | 120.55 | 38.23 | 40 | 8 |
| Haikou, Hainan | 12 | Non-Bt | 2014 | 42 | 110.62 | 19.91 | 5 | 37 |
| Anqing, Taihu | 13 | Bt | 2015 | 24 | 116.38 | 30.49 | 11 | 13 |
| Baoding,Hebei | 14 | Bt | 2015 | 24 | 115.14 | 38.83 | 13 | 11 |
| Chizhou, Anhui | 15 | Bt | 2015 | 24 | 116.99 | 30.12 | 20 | 4 |
| Dezhou, Shandong | 16 | Bt | 2015 | 72 | 115.97 | 36.97 | 72 | 0 |
| Handan, Hebei | 17 | Bt | 2015 | 24 | 115.27 | 36.53 | 22 | 2 |
| Jinzhong,Shanxi | 18 | Bt | 2015 | 36 | 112.38 | 37.39 | 36 | 0 |
| Jiujiang, Jiangxi | 19 | Bt | 2015 | 18 | 116.15 | 29.74 | 12 | 6 |
| Korla, Xinjiang† | 20 | Bt | 2015 | 48 | 86.12 | 41.75 | 20 | 28 |
| Shenyang, Liaoning | 21 | Bt | 2015 | 48 | 123.71 | 41.88 | 45 | 3 |
| Shihezi, Xinjiang | 22 | Bt | 2015 | 48 | 86.04 | 44.37 | 32 | 16 |
| Taizhou, Jiangsu | 23 | Bt | 2015 | 42 | 119.87 | 32.77 | 38 | 4 |
| Tangshan,Hebei | 24 | Bt | 2015 | 24 | 118.69 | 39.74 | 19 | 5 |
| Tongling, Anhui | 25 | Bt | 2015 | 24 | 118.01 | 30.97 | 23 | 1 |
| Weinan, Shanxi | 26 | Bt | 2015 | 24 | 109.97 | 34.83 | 24 | 0 |
| Xinxiang,Henan | 27 | Bt | 2015 | 24 | 113.88 | 35.06 | 23 | 1 |
| Xinxiang,Henan† | 28 | Bt | 2015 | 24 | 113.81 | 35.16 | 22 | 2 |
| Changde, Hunan | 29 | Bt | 2015 | 48 | 111.54 | 28.95 | 6 | 42 |
| Guangzhou, Guangdong† | 30 | Non-Bt | 2015 | 14 | 113.52 | 23.52 | 0 | 14 |
| Guilin, Guangxi | 31 | Non-Bt | 2015 | 14 | 110.47 | 25.27 | 5 | 9 |
| Sanya, Hainan | 32 | Non-Bt | 2015 | 24 | 109.53 | 18.22 | 3 | 21 |
| Baoding, Hebei | 33 | Bt | 2016 | 20 | 115.14 | 38.83 | 20 | 0 |
| Cangzhou, Hebei | 34 | Bt | 2016 | 24 | 116.89 | 38.3 | 22 | 2 |
| Langfang, Hebei | 35 | Bt | 2016 | 36 | 116.61 | 39.52 | 35 | 1 |
| Luohe, Henan | 36 | Bt | 2016 | 24 | 113.95 | 33.6 | 12 | 12 |
| Ruicheng, Shanxi | 37 | Bt | 2016 | 72 | 110.69 | 34.69 | 69 | 3 |
| Shangqiu, Henan | 38 | Bt | 2016 | 20 | 115.67 | 34.43 | 15 | 5 |
| Shenyang, Liaoning | 39 | Bt | 2016 | 23 | 123.71 | 41.88 | 21 | 2 |
| Taian, Shandong | 40 | Bt | 2016 | 24 | 117.1 | 36.17 | 19 | 5 |
| Taizhou, Jiangsu | 41 | Bt | 2016 | 24 | 119.87 | 32.77 | 20 | 4 |
| Wanrong, Shanxi | 42 | Bt | 2016 | 24 | 110.8 | 35.42 | 23 | 1 |
| Weinan, Shanxi | 43 | Bt | 2016 | 24 | 109.97 | 34.83 | 20 | 4 |
| Xinxiang,Henan | 44 | Bt | 2016 | 36 | 113.88 | 35.06 | 30 | 6 |
| Xinxiang,Henan† | 45 | Bt | 2016 | 24 | 113.81 | 35.16 | 22 | 2 |
| Yueyang, Shanxi | 46 | Bt | 2016 | 24 | 113.23 | 29.32 | 21 | 3 |
| Guangzhou, Guangdong | 47 | Non-Bt | 2016 | 23 | 113.52 | 23.52 | 10 | 13 |
| Guilin, Guangxi | 48 | Non-Bt | 2016 | 13 | 110.47 | 25.27 | 6 | 7 |
| Guiyang, Guizhou | 49 | Non-Bt | 2016 | 24 | 106.74 | 26.58 | 12 | 12 |
| Shenzhen, Guangdong | 50 | Non-Bt | 2016 | 36 | 114.05 | 22.58 | 13 | 23 |

† indicates larvae samples, otherwise all samples are adult moths

**Supplementary file 1h. Host information and accessions for samples.**

| Sample | Accession | Description |
| --- | --- | --- |
| Y24_1 | SRR11961703 | Cotton bollworm larvae sampled at 24h post-hatch |
| Y24_2 | SRR11961702 |  |
| Y24_3 | SRR11961693 |  |
| Y48_1 | SRR11961692 | Cotton bollworm larvae sampled at 48h post-hatch |
| Y48_2 | SRR11961691 |  |
| Y48_3 | SRR11961690 |  |
| Y72_1 | SRR11961689 | Cotton bollworm larvae sampled at 72h post-hatch |
| Y72_2 | SRR11961688 |  |
| Y72_3 | SRR11961687 |  |
| D24_1 | SRR11961686 | Cotton bollworm larvae sampled at 24h after HaDV2 infection |
| D24_2 | SRR11961701 |  |
| D24_3 | SRR11961700 |  |
| D48_1 | SRR11961699 | Cotton bollworm larvae sampled at 48h after HaDV2 infection |
| D48_2 | SRR11961698 |  |
| D48_3 | SRR11961697 |  |
| D72_1 | SRR11961696 | Cotton bollworm larvae sampled at 72h after HaDV2 infection |
| D72_2 | SRR11961695 |  |
| D72_3 | SRR11961694 |  |
| DVnBtn1 | SRR12000243 | HaDV2-negative cotton bollworm larvae fed on the artificial diet without Cry1Ac toxin |
| DVnBtn2 | SRR12000242 |  |
| DVnBtn3 | SRR12000239 |  |
| DVnBtp1 | SRR12000238 | HaDV2-negative cotton bollworm larvae fed on the artificial diet containing Cry1Ac toxin |
| DVnBtp2 | SRR12000237 |  |
| DVnBtp3 | SRR12000236 |  |
| DVpBtn1 | SRR12000235 | HaDV2-positive cotton bollworm larvae fed on the artificial diet without Cry1Ac toxin |
| DVpBtn2 | SRR12000234 |  |
| DVpBtn3 | SRR12000233 |  |
| DVpBtp1 | SRR12000232 | HaDV2-positive cotton bollworm larvae fed on the artificial diet containing Cry1Ac toxin |
| DVpBtp2 | SRR12000241 |  |
| DVpBtp3 | SRR12000240 |  |
